# Genetic diversity and phylogenetic analyses of Asian lineage Zika virus whole genome sequences derived from *Culex quinquefasciatus* mosquitoes and urine of patients during the 2020 epidemic in Thailand

**DOI:** 10.1038/s41598-023-45814-9

**Published:** 2023-10-27

**Authors:** Atchara Phumee, Suwalak Chitcharoen, Nataya Sutthanont, Proawpilart Intayot, Supaporn Wacharapluesadee, Padet Siriyasatien

**Affiliations:** 1 Department of Medical Technology, School of Allied Health Sciences, Walailak University, Nakhon Si Thammarat, Thailand; 2 Excellent Center for Dengue and Community Public Health (EC for DACH), Walailak University, Nakhon Si Thammarat, Thailand; 3 Department of Microbiology, Faculty of Medicine, Khon Kaen University, Khon Kaen, Thailand; 4 Department of Medical Entomology, Faculty of Tropical Medicine, Mahidol University, Bangkok, Thailand; 5 Pharmaceutical Ingredient and Medical Device Research Division, Research Development and Innovation Department, The Government Pharmaceutical Organization, Bangkok, Thailand; 6 Thai Red Cross Emerging Infectious Diseases Clinical Center, King Chulalongkorn Memorial Hospital, Bangkok, Thailand; 7 Center of Excellence in Vector Biology and Vector Borne Diseases, Department of Parasitology, Faculty of Medicine, Chulalongkorn University, Bangkok, Thailand

**Keywords:** Molecular biology, Infectious-disease diagnostics, Virology

## Abstract

Zika virus (ZIKV), a mosquito-borne flavivirus, has been continually emerging and re-emerging since 2010, with sporadic cases reported annually in Thailand, peaking at over 1000 confirmed positive cases in 2016. Leveraging high-throughput sequencing technologies, specifically whole genome sequencing (WGS), has facilitated rapid pathogen genome sequencing. In this study, we used multiplex amplicon sequencing on the Illumina Miseq instrument to describe ZIKV WGS. Six ZIKV WGS were derived from three samples of field-caught *Culex quinquefasciatus* mosquitoes (two males and one female) and three urine samples collected from patients in three different provinces of Thailand. Additionally, successful isolation of a ZIKV isolate occurred from a female *Cx. quinquefasciatus*. The WGS analysis revealed a correlation between the 2020 outbreak and the acquisition of five amino acid changes in the Asian lineage ZIKV strains from Thailand (2006), Cambodia (2010 and 2019), and the Philippines (2012). These changes, including C-T106A, prM-V1A, E-V473M, NS1-A188V, and NS5-M872V, were identified in all seven WGS, previously linked to significantly higher mortality rates. Furthermore, phylogenetic analysis indicated that the seven ZIKV sequences belonged to the Asian lineage. Notably, the genomic region of the E gene showed the highest nucleotide diversity (0.7–1.3%). This data holds significance in informing the development of molecular tools that enhance our understanding of virus patterns and evolution. Moreover, it may identify targets for improved methods to prevent and control future ZIKV outbreaks.

## Introduction

Zika virus (ZIKV) is an emerging RNA flavivirus transmitted by mosquitoes and is closely related to yellow fever virus (YFV), Japanese encephalitis (JEV), dengue virus (DENV), and West Nile virus (WNV)^1^. The ZIKV genome spans approximately 10.8 kb in length and comprises a positive-sense, single-stranded RNA molecule^2^. The genome organization of flaviviruses follows the sequence 5′-C-prM-E-NS1-NS2a-NS2b-NS3-NS4a-2K-NS4b-NS5-3′^3,4^. The structural components of the genome include the capsid protein (C), precursor membrane protein (Pr/M), and envelope protein (E). In addition, seven non-structural proteins (NS) play a critical role in genome replication^5^.

Recently, ZIKV has experienced rapid global spread and has been associated with human diseases such as microcephaly and other birth defects in neonates, as well as Guillain–Barré syndrome in adults^6,7^. ZIKV is mainly transmitted through mosquito vectors, predominantly *Aedes* mosquitoes^8^. However, ZIKV can also be transmitted by other routes^9^, including perinatal vertical transmission from mother to fetus^10^, blood transfusion^11^, and sexual transmission^12^. Sporadic cases of ZIKV have been reported annually in Thailand since 2010, mainly during the annual rainy season^13^. The rapid spread of ZIKV to 51 countries, including Thailand^14^, occurred between 2016 and 2017, with the Thai Ministry of Public Health recording over 1698 confirmed cases of ZIKV infection (1121 cases in 2016 and 577 cases in 2017)^13^. Previous studies in Thailand have identified ZIKV RNA in mosquitoes, including *Aedes aegypti*, *Culex quinquefasciatus*, and *Armigeres subalbatus*, collected from the homes of actively infected ZIKV patients^15,16^. In 2018, WGS was conducted on two ZIKV isolates from the blood of two Thai patients, showing genetic similarities to isolates from ZIKV outbreaks in Brazil during 2015–2016^17^. A subsequent report revealed that ZIKV has been circulating in Thailand since around 2002 and has significantly spread^18^. This conclusion was determined from an analysis of the spatial and age-related distribution of cases, along with the construction of time-resolved phylogenetic trees using genetic data from Thailand and other areas^18^. Following a decline in the number of ZIKV cases from 273 cases in 2019 to 239 cases in 2020, and 63 cases in 2021, there has been a recent increase in cases, reaching 190 cases in 2022 and 479 cases in 2023^13^. This resurgence highlights the urgency of studying the severity of diseases caused by ZIKV. The virus’s rapid spread necessitates an immediate and comprehensive understanding to address the emerging public health concerns.

For ZIKV diagnosis, the recommended gold standard is molecular-based detection of ZIKV RNA in samples, employing methods such as reverse transcription PCR (RT-PCR), real time RT-PCR and nested RT-PCR assay across several alternative specimen types^19^.The recent emergence of high-throughput sequencing technologies, notably whole genome sequencing (WGS), now enables the rapidly acquisition of complete pathogen genomes^20^. Phylogenetic analyses play a crucial role in elucidating the evolutionary dynamics and dissemination patterns of ZIKV, with two major lineages recognized as the African and Asian lineages based on their initial discoveries in the respective geographic regions^2,21^. Examining genetic diversity among ZIKV lineages and across the genome offers valuable insights into the current genotype of the circulating virus^22^. Several investigations have reported nucleotide (nt) mutations and amino acid (aa) changes throughout the entire diversity of ZIKV^23–25^. The presence of specific mutations in all sequences of a specific clade in the phylogeny may hold significance for the control of disease, especially if that clade is associated with increased pathogenicity. However, it is crucial to validate the causal relationship between a mutation and a phenotype through reverse genetics.

Therefore, our objective was to characterize ZIKV genetic diversity and perform phylogenetic analyses of positive ZIKV samples through complete genome sequence using multiplex amplicon sequencing-based next-generation sequencing (NGS) on an Illumina sequencing system (MiSeq). Scaling ZIKV whole-genome sequencing presents a valuable opportunity to understand the genetic diversity, evolutionary dynamics, and transmission patterns of ZIKV. The insights gained from this study can be applied to further develop of new molecular tools, and the nucleotide sequence data can contribute to the future development of more effective, region-specific prevention, diagnostics, and control strategies for ZIKV in Thailand.

## Results

### ZIKV detection and isolation

**Figure 1:**
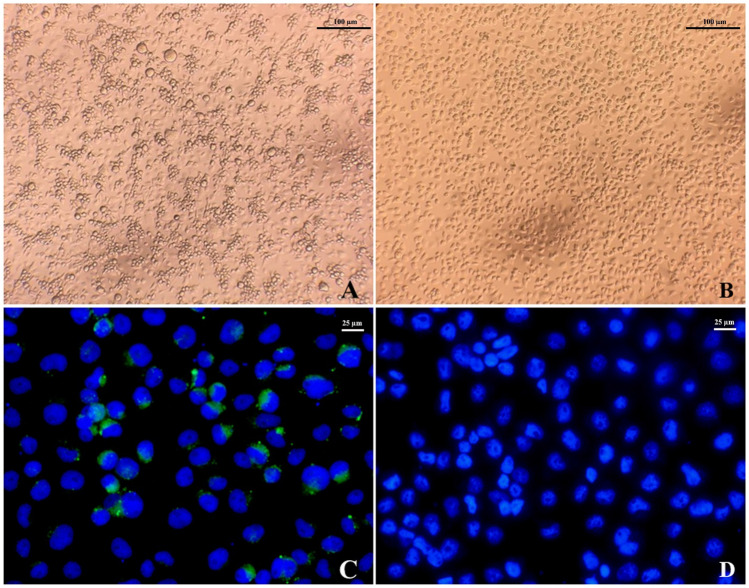
The isolation of ZIKV from *Cx. quinquefasciatus* in C6/36 cells. The CPE in infected cells at 7 dpi (**A**) and uninfected cells (**B**) (100× magnification). The immunofluorescence assay (IFA) was used to detect ZIKV antigens in C6/36 cells at 7 dpi (**C**) and uninfected cells (**D**) (400× magnification); Green fluorescent color indicates viral replication; DAPI stains cell nuclei (blue fluorescent color). Photos taken by Atchara Phumee, First author.

In 2020, a total of 529 male and female mosquitoes, comprising *Cx. quinquefasciatus* (n = 326), *Ae. aegypti* (n = 154)*, Ae. albopictus* (n = 10), and *Ar. subalbatus* (n = 39), were collected from the residences of active ZIKV patients in Bangkok, Chanthaburi, and Nakhon Ratchasima provinces. Hemi-nested RT-PCR (hn-RT-PCR) was employed to screen the presence of ZIKV in both mosquitoes and urine samples from patients with active ZIKV infection. ZIKV was detected in 29 out of 529 samples (5.5%), distributed across *Cx. quinquefasciatus* (13 males and 10 females), *Ae. aegypti* (2 males and 3 females) and *Ar. subalbatus* (1 male) (Table 1). Primary detection of ZIKV positive samples by hn-RT-PCR was performed by complete genome sequence. Additionally, the supernatants of all hn-RT-PCR-positive mosquito samples were used to isolate ZIKV by inoculation onto C6/36 cells. Successful isolation of ZIKV was achieved from one naturally-infected female *Cx. quinquefasciatus* mosquito sample in the C6/36 cells, collected from Nakhon Ratchasima province. At 7 days post-infection (dpi), cytopathic effects (CPE) can be observed in one sample of ZIKV isolates derived from mosquitoes (Fig. 1A). The prominent morphological characteristics of CPE included a random-packed cell structure, damaged cell membranes, and lysis, while no morphological changes were observed in the normal monolayer of the C6/36 cell control under light microscopy (Fig. 1B). Furthermore, a ZIKV-specific immunofluorescence assay (IFA) staining was performed to determine the location of the ZIKV antigen. DAPI stain was used to locate the nucleus of each cell. Green fluorescent dots corresponding to the antibody with ZIKV antigen were observed in the cytoplasm of cells infected at 7 dpi (Fig. 1C). In contrast, no green fluorescent signal was detected in infected cells at 0, 3, and 5 dpi, nor at any timepoint in uninfected control C6/36 cells (Fig. 1D).

**Table 1:** ZIKV detection in mosquitoes collected from in Bangkok, Chanthaburi, and Nakhon Ratchasima provinces of Thailand.

Province	ZIKV Positive sample (positive/total samples)								Successfully cultured
	*Cx. quinquefasciatus*		*Ae. aegypti*		*Ae. albopictus*		*Ar. subalbatus*		
	Male	Female	Male	Female	Male	Female	Male	Female	
Bangkok	8/79	4/102	1/50	2/35	–	–	–	–	
Nakhon Ratchasima	1/31	4/47	0/18	0/14	–	0/10	0/4	0/7	1 sample of female *Cx. quinquefasciatus*
Chanthaburi	4/39	2/28	1/13	1/24	–	–	1/4	0/24	
Total	13/149	10/177	2/81	3/73	0/0	0/10	1/8	0/31	
	29/529

### Genomic diversity of complete genome sequences of ZIKV

**Figure 2:**
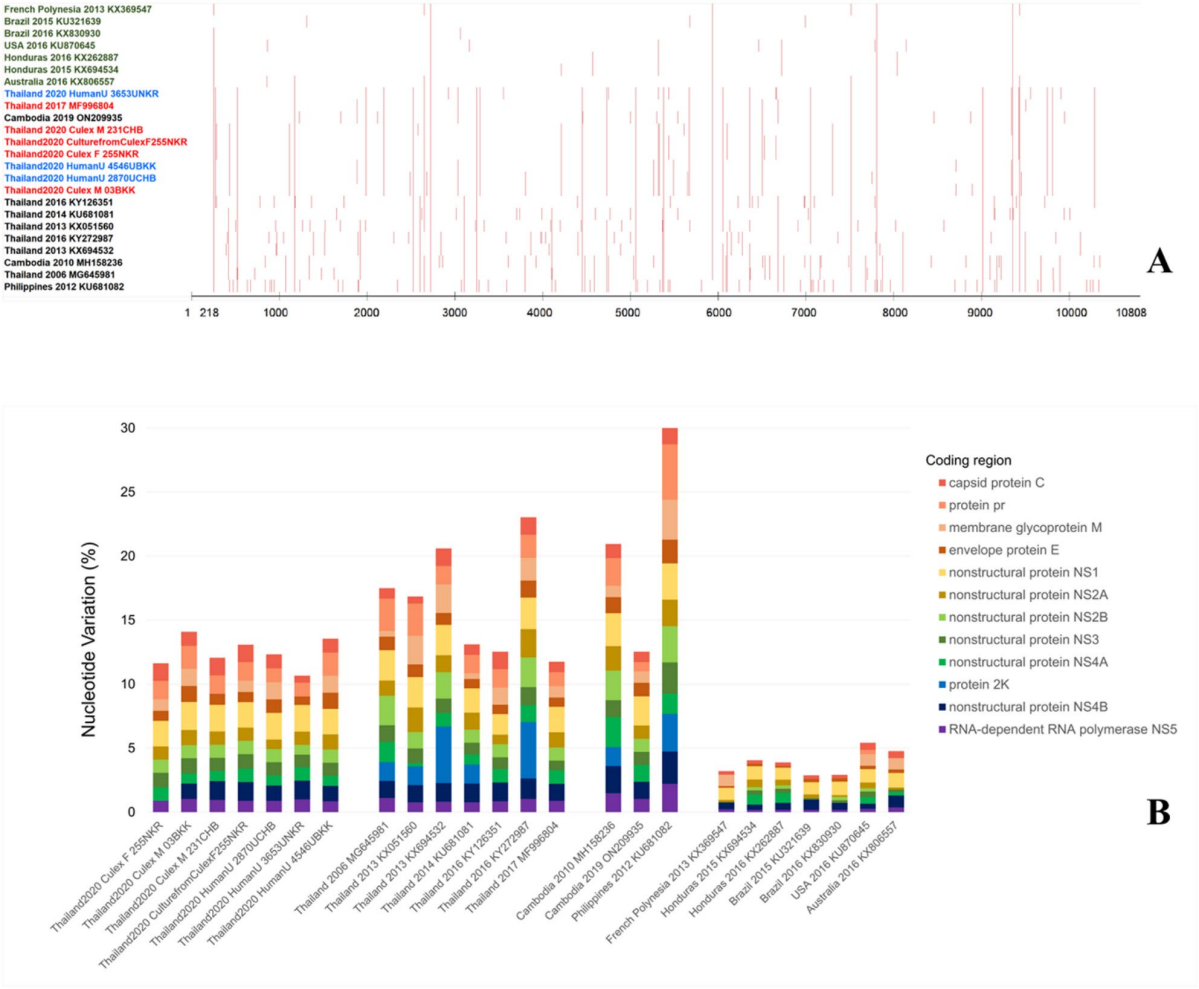
Analysis of nucleotide variation in each sample based on the number of consensus nucleotides (**A**) and the percentage of average nucleotide variation per gene in the study, Asia, and non-Asia ZIKV sequences (**B**). All figures were modified from free software under public domain or a free license.

**Figure 3:**
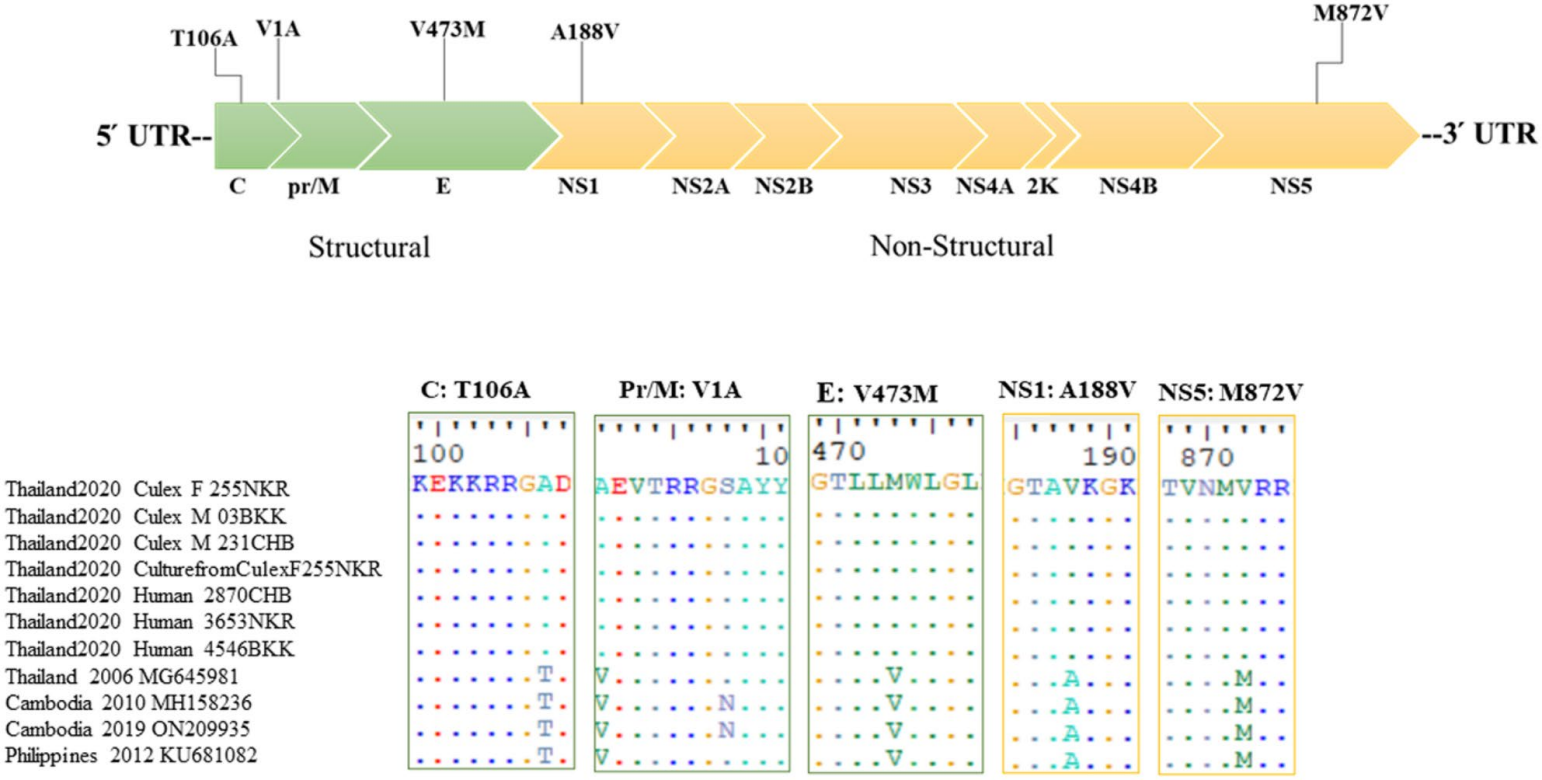
The five mutation-mediated evolutionary adaptation of ZIKV isolates belonging to the Asian lineage. All figures were modified from free software under public domain or a free license.

Samples yielding positive hn-RT-PCR results comprised three ZIKV positive mosquito samples, one ZIKV isolated from mosquito, and three urine samples, all exhibiting sufficient quality for complete genome sequence on an Illumina NGS instrument. Consequently, seven sequences of complete genome sequence were analyzed in this study. Post primer-trimming, alignment, variant calling, and consensus generation, the alignment of these seven samples was approximately 10,270 nucleotides (nt) in length. The ZIKV genome encodes three structural proteins: C (365 nt), Pr/M (502 nt), E (1511 nt), and seven non-structural proteins: NS1 (1055 nt), NS2A (677 nt), NS2B (389 nt), NS3 (1850 nt), NS4A (380 nt), 2 K (68 nt), NS4B (752 nt), and NS5 (2708 nt). The complete genome sequences of ZIKV have been deposited in GenBank under the accession numbers OR535163-OR535169. Sequence analysis of ZIKV revealed that our seven genome sequences were related to ZIKV sequences isolated from the serum of patients in Thailand during the outbreak of ZIKV in 2006–2017, Cambodia in 2010 and 2019 (accession no. MH158236.1 and ON209935.1), and the Philippines in 2012 (accession no. KU681082.1) (Fig. 2A). Nucleotide variation evaluation was conducted for each sample, which the average count and proportion of minority variants determined in relation to the reference sequence from Brazil (accession no. NC035889.1). The seven ZIKV sequences showed nucleotide variation compared to ZIKV derived from both patient and mosquito samples. The ZIKV nucleotide variation between patient and mosquito showed an approximate variation of 0.4% from Bangkok, 0.6% from Nakhon Ratchasima, and 0.8% from Chanthaburi. ZIKV cultured from female mosquito from Nakhon Ratchasima exhibited complete genetic similarity (100%) to the mosquitoes collected from Nakhon Ratchasima, and 0.6% to the ZIKV present in the infected patient from Nakhon Ratchasima. Overall, nucleotide variation was observed, ranging from 0.5 to 0.8% in patients and 0.4–0.9% in mosquitoes. The E region demonstrated the highest nucleotide diversity, ranging from 0.7 to 1.3%. The non-structural proteins (NS1, NS2B, NS3, NS4A, NS4B, and NS5) showed low nucleotide diversity, approximately 0.2–0.3%, while the NS2A region showed higher nucleotide diversity compared to other NS regions (0.5%). The percentage of nucleotide variation indicated minor variations in non-Asian-lineage ZIKV belonging to the Asian lineage of ZIKV (Fig. 2B). In the amino acids study, several changes were observed in all seven of our ZIKV genome sequences. Most of these changes were previously reported in ZIKV isolates from Thailand, Cambodia, and the Philippines, including C-T106A, prM-V1A, E-V473M, NS1-A188V, and NS5-M872V (Fig. 3). However, these five mutations were not observed in ZIKV isolates collected in Thailand during the 2013–2017 epidemic. Instead, these mutations were detected in Thailand in 2006, Cambodia in 2010 and 2019, and the Philippines in 2012.

### Phylogenetic analysis based on the complete genome sequence of ZIKV

**Figure 4:**
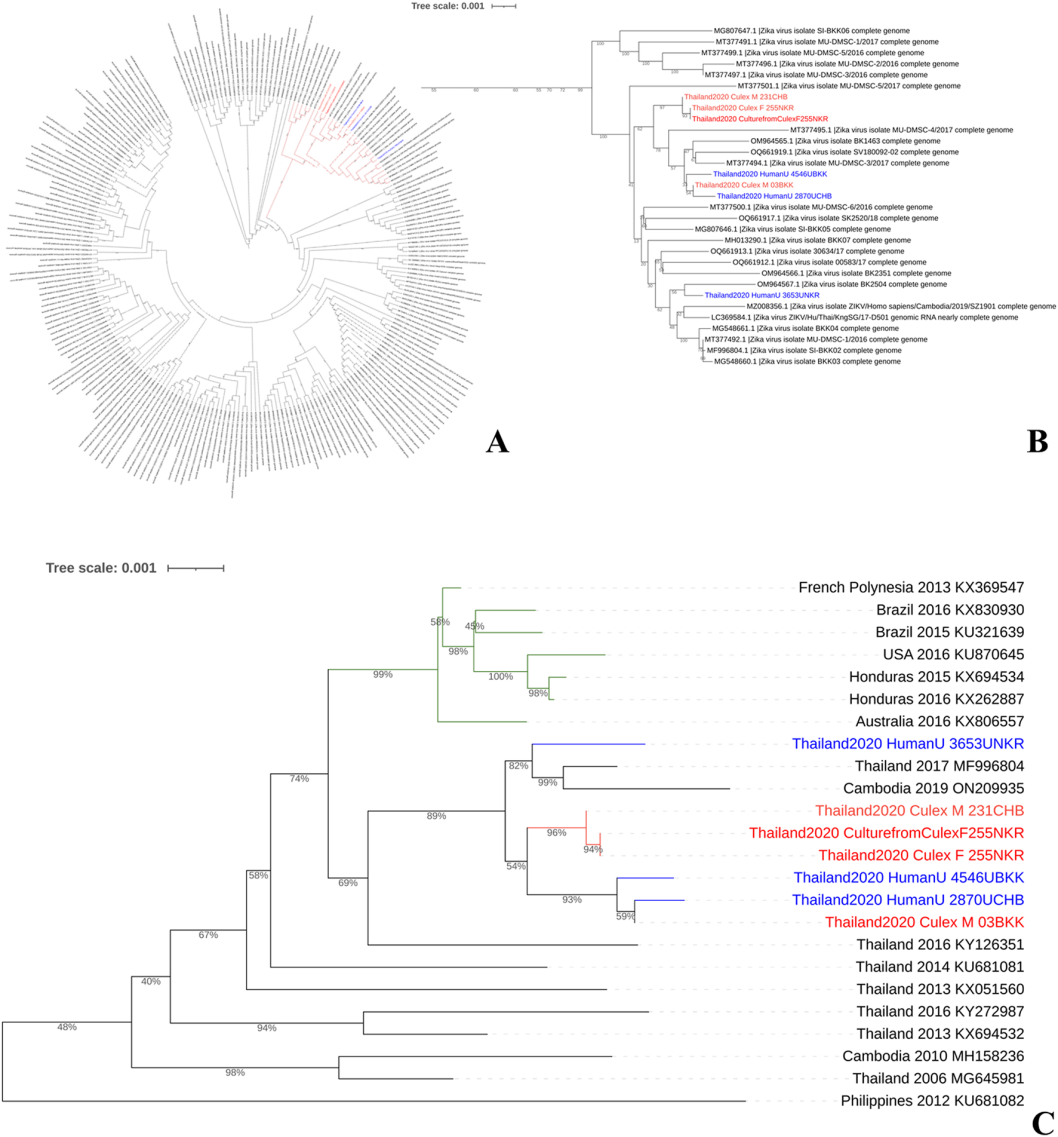
Phylogenetic analysis of 224 ZIKV complete nucleotide sequences isolates obtained from the NCBI Virus database worldwide in 2000–2023 (**A**), expansion clade of tree containing ZIKV sequence in this study (**B**), and the maximum likelihood (ML) phylogenetic based on ZIKV isolates from Asia and non-Asia regions (**C**). The red and blue colors indicate the mosquito and human samples, respectively. The green color denotes the ZIKV non-Asia isolates. All figures were modified from free software under public domain or a free license.

To determine the genetic relationship between the ZIKV isolates obtained in this study and other ZIKV strains collected from various geographical locations, we constructed a phylogenetic tree using complete genome sequences. Our seven genome sequences underwent alignment analysis with 224 full-length ZIKV isolates derived from 29 distinct countries between 2000 and 2023, as established in the GenBank database. The phylogenetic analysis revealed that the seven ZIKV isolates belonged to the Asian lineage and showed a close relationship with ZIKV strains obtained from human blood samples in Thailand from 2017 to 2019, as well as from a human saliva sample in Cambodia in 2019 (Fig. 4A,B). Moreover, we reconstructed another phylogenetic tree for evolutionary distances using the neighbor-joining method and a maximum composite likelihood algorithm. The ZIKV genomes sequenced in this study are found in two separate clusters within a clade, alongside genomes from Thailand in 2017 and Cambodia in 2019. However, in our genomic analyses, the non-Asia sequences formed a monophyletic clade distinct from the Asia sequences. Additionally, all ZIKV sequences from mosquitoes and patients in different regions showed that the branch lengths of the patients tend to be longer than those observed in mosquitoes (Fig. 4C).

## Discussion

Zika virus infection has historically been a neglected viral infection in Africa, the Americas, Asia, and the Pacific, likely due to the prevalence of silent infections and relatively mild clinical manifestations^26,27^. Southeast Asian countries such as Thailand^28^, Cambodia^29,30^, Indonesia^31^, the Philippines^32^, India^33^, Singapore^34^, Japan^35^, and Vietnam^36,37^ have experienced continuous circulation of ZIKV. Thailand, a geographically diverse country and popular tourist destination, has seen cases of ZIKV infection among travelers to various regions, including Japan^38^, Europe^39^, Israel^40^, and Canada^41^. Despite a significant increase in the incidence of ZIKV infection in the past year, the overall situation remains unchanged, posing a potential public health concern in Thailand. The primary approach to preventing and controlling ZIKV infection is vector control.

This current study reports the detection and isolation of ZIKV in field-caught mosquitoes and the urine of patients in Thailand during part of the 2020 epidemic. ZIKV was found in both male and female *Cx. quinquefasciatus* and human urine. The detection of ZIKV RNA in two males *Cx. quinquefasciatus* suggests the occurrence of vertical transmission of ZIKV in this species*.* Phumee et al.^16^ previously demonstrated that ZIKV could be vertically transmitted in female and male laboratory colonies of *Cx. quinquefasciatus* until the F_6_ and F_2_ generations, respectively. Nevertheless, the study found that ZIKV was only detected in the F_1_ generation of both male and female *Ae. aegypti*, and *Ae. albopictus* were unable to vertically transmit ZIKV^16^.

This study marks the first report of complete genome sequences of ZIKV obtained from *Cx. quinquefasciatus* in Thailand. The seven ZIKV sequences in this study exhibited good coverage, exceeding 99.83–100%. To better understand the evolutionary relationships between these sequences, we constructed phylogenetic trees using the ZIKV genomes. These ZIKV isolates formed strong clustering with ZIKV complete genome sequences obtained from other patients in Thailand during the ZIKV outbreak in 2006–2017, Cambodia in 2010 and 2019, and the Philippines in 2012. It is most likely that ZIKV initially spread among neighboring countries in Southeast Asia, including Cambodia, which shares a border in the east of Thailand. An interesting observation was the longer branch lengths of the patients compared to those infecting mosquitoes. This suggests a potential host-specific adaptation, with the patients accumulating more genetic mutations. Furthermore, examining data from the Thai population revealed that the branch length for the year 2020 was the longest, indicating ongoing viral evolution and genetic variation within this region. This implies that factors such as host switching and time may exert selection pressures on ZIKV. However, it is essential to consider that branch lengths alone may not provide a complete understanding of virus evolution, and complementary analyses are necessary for a comprehensive assessment of viral dynamics.

Our study demonstrated five mutations in all seven ZIKV genome, including C-T106A, prM-V1A, E-V473M, NS1-A188V, and NS5-M872V. Zhang et al.^42^ revealed that a novel ZIKV isolate from Cambodia in 2019 (CAM/2019), showcasing 12 amino acid mutations when compared to Cambodia in 2010 (CAM/2010). These mutations include C‐T106A, prM‐V123A, prM‐N130S, prM‐M151L, E‐V763M, NS1‐A982V, NS2A‐A1204T, NS2A‐P1274L, NS2B‐A1477T, NS5‐V2878A, NS5‐M3392V, and NS5‐V3403M. Notably, some of these mutations were associated with neurovirulence^42^. Furthermore, the CAM/2019 virus showed an NS2A-A1204T mutation closely related to the Thai isolate SI-BKK02 (accession no. MF996804.1), collected from Thailand in 2017 and linked to a case of microcephaly^43^. Amino acid changes reported in Asian-lineage ZIKV strains from 2010 to 2013 encompassed C-T106A, prM-V1A, prM-S17N, E-V473M, NS1-A188V, NS5-M114V, and NS5-M872V^44–47^. Yu et al.^48^ discovered that the C-T106A mutation heightened infectivity and accelerated the spread of ZIKV in both mosquitoes and rodents, enhancing transmissibility between vectors and hosts. The NS1-A188V mutation exhibited a modest impact on ZIKV infectivity in laboratory *Ae. aegypti* mosquitoes. Additionally, this mutation was found to inhibit the production of type I interferon (IFN) in mammals^44,45^. According to Shan et al.^49^, mutants C-T106A, E-V473M, and NS5-M872V associated with significantly elevated mortality rates. In particular, the E-V473M mutation was identified to amplify ZIKV replication, resulting in enhanced neurovirulence, increased transmission from mother to fetus during pregnancy, and the presence of viremia in both mouse and non-human primate models^49^.

In this study, we conducted a comparative analysis of the genetic diversity offered by ZIKV mutants containing distinct stable substitutions that emerged within the Asian ZIKV lineage. Genetic variation can provide crucial insights into ZIKV biology and pathogenesis, potentially uncovering functional mutations in the virus. However, the potential relevance of these mutations in this study remains uncertain. The study is limited by a relatively small sample size, encompassing only seven complete genomes collected from different geographical regions in Thailand. The sample collection methodology relied on a report from the Department of Disease Control, Ministry of Public Health, which tested blood samples for ZIKV RNA presence. In general, we collected the mosquitoes in and around the residences of active ZIKV patients whose blood tested positive for ZIKV RNA. Consequently, upon field arrival, we could collect only non-invasive samples such as urine from patients in each area. Moreover, research suggests that ZIKV detection in urine is more sensitive and has a longer window of detection compared to serum and saliva^50,51^. These considerations elucidate why our study exclusively focused on urine samples.

To comprehensively assess the transmissibility of mutant ZIKV strains within mosquitoes, further investigation using a mosquito-mouse transmission model is necessary. This study not only establishes a scientific basis for understanding the evolution of the ZIKV in Southeast Asia but also illustrates the importance of continued monitoring in future outbreaks of ZIKV. Emphasizing diagnostic challenges, surveillance, and implementing effective control and prevention strategies for ZIKV becomes paramount in stopping the rapid spread of the epidemic in the future.

## Methods

### Sample collection

Adult mosquitoes were collected both inside and outside the residences of ZIKV-infected patients during the 2020 ZIKV outbreaks in Bangkok, Chanthaburi, and Nakhon Ratchasima using a backpack aspirator (Bioquip, CA, USA). All mosquitoes were classified according to their sex and species using morphological identification. Urine samples from ZIKV-infected patients were collected. Subsequent testing for ZIKV infection was conducted on both individual mosquitoes and human samples.

### ZIKV RNA detection by hn-RT-PCR

Individual mosquitoes were placed in 1.5 ml microcentrifuge tubes with 200 µl of 1X phosphate buffered saline (PBS), ground, and centrifuged at 13,000×*g* for 10 min. Then, 200 µl of the supernatant was transferred to a sterile 1.5 ml microcentrifuge tube mixed with 200 µl of 2X minimum essential medium Eagle (MEM) (HyClone, USA) containing 2% heat-inactivated FBS (Gibco, USA), 2% penicillin (100 U/ml), and streptomycin (100 µg/ml) (Sigma-Aldrich, USA), and stored at − 80 °C for further virus isolation attempts. The mosquito carcass or human urine sample was mixed with 300 µl of lysis buffer and processed for viral RNA extraction using an Invisorb Spin Virus RNA Mini Kit (STRATEC Molecular GmbH, Germany). The mosquito or human urine RNA samples were amplified for detection of ZIKV at NS5 gene using hn-RT-PCR (Thai Patent No. 2001004011, 2020)^15^. The positive control was constructed and used the lower band of a synthetic positive control plasmid. The amplified PCR products were separated on a 1.5% agarose gel, stained with ethidium bromide, and visualized using Quantity One Quantification Analysis Software Version 4.5.2, Gel Doc EQ System (Bio-Rad, USA).

### Virus isolation and propagation

The supernatant from ZIKV-PCR positive samples was passed through 0.2 µm syringe filters and seeded onto monolayers of *Ae. albopictus* C6/36 cells in 12-well plates, then gently mixed at room temperature for 1 h. The supernatant was removed and replaced with 2 ml of MEM containing 1% heat-inactivated FBS (Gibco, USA), penicillin (100 U/ml), and streptomycin (100 µg/ml) (Sigma-Aldrich, USA). The plates were incubated at 28 °C and 5% CO_2_. CPE was monitored daily for 6–7 days. The hn-RT-PCR amplification of CPE-positive culture supernatant on day 7 confirmed ZIKV infection. The positive isolates were propagated in C6/36 cells cultured in MEM with 10% heat-inactivated FBS (Gibco, USA) at 28 °C and 5% CO_2_ for three more passages. Harvested supernatant was kept at − 80 °C until use.

### Immunofluorescence assay (IFA)

At 0, 3, 5, and 7 days post-infection (dpi), infected cells grown on glass coverslips were harvested and rinsed with 1X PBS (3 × 5 min), fixed with 4% formaldehyde for 15 min at room temperature, and then incubated for 15 min in 0.1% of Triton X-100 in 1X PBS for 15 min at room temperature. After washing with 1X PBS (3 × 5 min), the fixed cells were incubated with a 1:500 primary rabbit-Zika virus NS1 protein antibody (GeneTex, USA), rinsed with 1X PBS (3 × 5 min), and incubated with 1:10,000 Goat Anti-Rabbit IgG H&L (Alexa Fluor 488) (Abcam, UK) at room temperature for 1 h as the secondary antibody and then washed with 1X PBS (3 × 5 min). Finally, a drop of Prolong gold antifade (Invitrogen, USA) was added on each slide and topped with a cover slide. The non-infected C6/36 cells were used as a negative control in the experiment. All slides were examined under a fluorescence microscope (Nikon, Japan).

### WGS using multiplex amplicon sequencing-based NGS

RNA from positive samples were synthesized for cDNA using Superscript IV enzyme (Invitrogen, USA) followed by ZIKV multiplex PCR of 2 primer pools, long‐amplicon 35‐plex PCR primer panel with ~ 11,000‐bp amplicon lengths for full‐genome sequencing of ZIKV^20^. The PCR products from 2 primer pools were purified and end-repaired using KAPA Hyper prep kit (Kapa Biosystems, USA). The index adaptor was then added to the end of the fragments and PCR was performed. The DNA library was checked for quality and quantity using QIAxcel screen gel (Qiagen, USA) and Qubit high sensitivity DNA kit (Thermo Fisher Scientific, USA), respectively. The concentration of each DNA library was calculated. The pooled DNA library was then loaded into the MiSeq reagent kit V3 (Illumina, USA) and sequenced using the MiSeq machine (Illumina, USA).

### Phylogenetic tree construction

The genome sequences were assembled using multiple alignments using fast Fourier transform (MAFFT) version 7 (https://mafft.cbrc.jp/alignment/server/)^52^. Based on the complete genome sequence of ZIKV from this study and reference strains obtained from the GenBank database. The phylogenetic trees were constructed using the neighbor-joining (NJ) method and maximum composite likelihood model, based on the general time reversible (GTR) model in MEGA 11 software^53^. Bootstrap values were estimated for 1000 replicates. The phylogenetic trees were edited using Interactive Tree Of Life (iTOL) v6.3^54^. Variation and mutation in the genome are confirmed by manually checking the alignment with ZIKV reference genome from Brazil (accession no. NC035889.1) in Unipro UGENE v47.0^55^.

### Ethics declarations

The study was approved by the animal research ethics committee of Chulalongkorn University and adhered to the Animal Care and Use Protocol (CU-ACUP). The Faculty of Medicine, Chulalongkorn University, Bangkok, Thailand (COA No. 025/2564) approved this study, which abided by the Animals for Scientific Purposes Act and all relevant institutional policies and regulations regarding animal care and use at Chulalongkorn University. The use of hazardous agents was only initiated after approval from the institutional animal care and use committee (IACUC), Institutional Biosafety Committee (IBC), and/or Environmental Health and Safety Department. The use of human blood was approved by the Institutional Review Board of the Faculty of Medicine, Chulalongkorn University, Bangkok, Thailand (COE No. 016/2017), and the study was conducted in compliance with the international guidelines for human research protection as stated in the Declaration of Helsinki, The Belmont Report, the Council for International Organizations of Medical Sciences (CIOMS) guidelines and the International Conference on Harmonization in Good Clinical Practice (ICH-GCP). Informed consent was obtained from all participants. All experimental protocols requiring biosafety were approved by Institutional Biosafety Committees (IBC) of the Faculty of Medicine, Chulalongkorn University, Bangkok, Thailand (MDCUIBC002/2017).

## Data Availability

The data that support the findings of this study are available from the corresponding author on reasonable request. The ZIKV-sequences are available in GenBank, accession number OR535163-OR535169.
